# 

**Published:** 1839

**Authors:** 


### BIBLIOGRAPHICAL RECORD.

1. The Danger, Irrationality, and Evils of Medical Quackery; also the Causes of Success, &c. By CHARLES COWAN, M.D. Physician to the Reading Dispensary. 8vo, pp. 38. With an Appendix, containing the Composition of some Quack Medicines.

2. An Inquiry into the Morbid Effects of Deficiency of Food, chiefly with reference to their Occurrence amongst the Destitute Poor; also Practical Observations on the Treatment of such Cases. By RICHARD BARON HOWARD, M.D. Physician to the Ardwick and Ancoats Dispensary, &c. 8vo, pp. 77. Simpkin and Co. 1839.

3. The Phrenological Journal, &c. No. 40, published quarterly. July, 1839.

4. Delirium Tremens, its Diagnoses, Causes, and Treatment. By JULIUS WOLF, M.D. Small 8vo, pp. 50. W. Pearson, H. Jackson.

5. The Wonders of Geology. By GIDEON ALGERNON MANTELL, LL.D. &c. In two volumes, 8vo, 3d Edition. London, Relfe and Fletcher, 1839.

*** *This Edition is a real treasure to the contemplative mind.*

6. Tables shewing the Temperature of Cove, for the Year 1838, and contrasting it with Torquay, Exeter, Cheltenham, &c. &c. By DAVID SCOTT, M.D.

7. First Annual Report of the Registrar-General of Births, Deaths, and Marriages in England. 4to, 1839.

8. Register of Experiments on Animals, anatomical, physiological, and pathological. By JAMES TURNER, Veterinary Surgeon, &c. Octavo, pp. 51. Longman and Co. 1839.

9. Dental Surgery. The American Journal of Dental Surgery, for June, 1839. Vol. I. No. 1. Edited by CHAPEN A. HARRIS, of Baltimore, and ELEAZER PARMLY, of New York.

10. A History of British Birds. By W. YARRELL, F.L.S. illustrated by Woodcuts, &c. Parts XIII. and XIV. Price 2s. 6d. J. Van Voorst. July, 1839.

11. A History of British Reptiles. By THOMAS BELL, F.R.S. &c. Part III. (completing the Work.) Price 3s. Van Voorst. July, 1839.

12. Medical Portrait Gallery, Part XVII. price 3s. monthly. By T. J. PETTIGREW, F.R.S. Contains Memoirs and Portraits of James Ware, F.R.S. and Sir Charles Bell, K.H. F.R.S. &c. Whittaker and Co. July, 1839.

13. The Physiognomy of Mental Diseases. By Sir A. MORRISON, M.D. No. 11, 12, & 13, price 3s. 6d. Monomania, with vicious propensities.

14. A Treatise on Diseases of the Heart and Great Vessels, and the Affections which may be mistaken for them, &c. &c. By J. HOPE, M.D. F.R.S. Physician to St. George's Hospital, &c. Third Edition, corrected and greatly enlarged. J. Churchill, London, July, 1839.

15. Memoirs of the Life of Sir Humphrey Davy, Bart. By his Brother, J. DAVY, M.D. Pp. 444. Vol 1. of the Author's Works. Smith, Elder and Co. July, 1839.

16. Physic and Physicians—a Medical Sketch-book, containing the public and pri vate life of the most celebrated Medical Men of former days; with Memoirs of eminent living London Physicians and Surgeons. In two volumes, 8vo. London, Longman and Co. 1839.

17. Human Physiology. For the Use of Elementary Schools. By CHARLES A. LEE, M.D. of New York. New York, 1838.

18. The Quarterly Journal of the Calcutta Medical and Physical Society, &c. Nos. V. and VI. Conducted by Drs. GOODEVE and O'SHAUGNESSY. Calcutta, Jan. and July, 1839.

19. The Surgical Anatomy of the Arteries, and Descriptive Anatomy of the Heart: together with the Physiology of the Circulation in Man and inferior Animals. By

Valentine Flood, A.M. M.D. T.C.D. Member of the Royal Irish Academy, &c. Octavo, pp. 237. Highley, London. July, 1839.

19. The Surgical Anatomy of the Groin, the Femoral and Popliteal Regions. By Thomas Merton, formerly one of the House-surgeons of University College Hospital. Illustrated with Lithographic Plates and Wood-engravings. Royal 8vo, pp. 207. Taylor and Walton, London, July, 1839.

20. A Treatise on the Medical Jurisprudence of Insanity. By J. Ray, M.D. 8vo, pp. 326. Clarke, Edinb. 1839.

21. Illustrations of the Comparative Anatomy of the Nervous System. By Joseph Swan. Part 5, price 7s. Longman and Co. 1839.

22. Report of the Directors of the Montrose Lunatic Asylum, Infirmary, and Dispensary, for the Year ending 1st of June, 1839.

23. A Practical Treatise on the Human Teeth: shewing the Causes of their Destruction, and the Means of their Preservation. By William Robertson. With Plates. Octavo, pp. 206. Hayward and Moore. Second Edition.

24. Medical Portrait Gallery. Part 18. By T. J. Pettigrew, Esq. Memoirs of Hippocrates and Dr. Bostock.

25. Nineteenth Annual Report of the Directors of the Dundee Royal Asylum for Lunatics, for the Year ending 31st May.

26. Medical Report of the Managers of the Lunatic Asylum of Aberdeen, for the Year ending 30th April, 1839.

27. Human Physiology for the Use of Elementary Schools. By Charles A. Lee, M.D. Professor of Materia Medica in the University of the City of New York. New York. 18mo. Pp. 224.

28. An Address to Medical and Surgical Pupils, on the Studies and Duties of their Profession; to which is appended a Case of Cæsarian Operation. By James Barlow, Surgeon, Blackburn. 8vo, pp. 119.

29. Observations on the Disorder of the General Health of Females, called Chlorosis; shewing the true Cause to be entirely independent of Peculiarities of Sex. By Samuel Fox, Surgeon. Octavo, pp. 132. London, 1839.

30. Retrospect of the Progress of Surgical Literature, for the Year 1838-9. By Messrs. Newnham, W. Wickham, and Salter. Price Two Shillings. London, 1839.

31. A System of Anatomical Plates, with Descriptive Letter-press. By John Lizars, F.R.S.E. &c. Edinburgh. Part I. Price 10s. 6d. coloured, or 7s. 6d. plain.

32. Narrative of the Discoveries of Charles Bell in the Nervous System. [illegible] Alexander Shaw, Assistant Surgeon [illegible] the Middlesex Hospital. Octavo, pp. 232. Longmans', 1839.

33. Practical Observations, showing th[illegible] Mercury is the sole Cause of what a[illegible] termed Secondary Symptoms. By P. Murphy, M.D. Licentiate of the Ro[illegible] College of Surgeons in Ireland. Pp. 10[illegible]

34. Documents and Dates of Mod[illegible] Discoveries in the Nervous System. Octav[illegible] London, Churchill, 1839.

35. A General Outline of the Anim[illegible] Kingdom, and Manual of Comparative An[illegible] tomy, &c. By T. R. Jones, F.Z.S. Part VI Price 2s. 6d. Van Voorst. London, Sep[illegible] 1839.

36. Laws of the Parisian Medical So[illegible] ciety. Established 1837.

37. Medical and Physiological Problem[illegible] being chiefly Researches for correct Prin[illegible] ciples, &c. By W. and D. Griffin, M.[illegible] Part I. Limerick, 1839.

38. Inaugural Dissertation on the Phy[illegible] siological Inferences to be deduced fr[illegible] the Structure of the Nervous System, & By W. B. Carpenter, M.D. Edinb. 183[illegible] Churchill, London.

39. The New York Journal of Medicin[illegible] and Surgery. Quarterly. No. 1. July 1839. New York. Churchill, London 1839.

40. Early Miscellaneous Papers fr[illegible] 1799 to 1805. With an Introductory Le[illegible] ture and Outlines of Lectures on Chemistry, delivered in 1802 and 1804. By Sir Humphry Davy, Bart. Vol. II. Smith and Elder, 1839.

